# GMO Genetic Elements Thesaurus (GMO-GET): a controlled vocabulary for the consensus designation of introduced or modified genetic elements in genetically modified organisms

**DOI:** 10.1186/s12859-020-03880-0

**Published:** 2021-02-05

**Authors:** Paulien Adamse, Emilie Dagand, Karen Bohmert-Tatarev, Daniela Wahler, Manoela Miranda, Esther J. Kok, Joachim Bendiek

**Affiliations:** 1 Wageningen Food Safety Research (WFSR), PO Box 230, 6700 AE Wageningen, Netherlands; 2 Federal Office of Consumer Protection and Food Safety, PO Box 11 02 60, 10832 Berlin, Germany; 3 Yield10 Bioscience, Inc., 19 Presidential Way, Woburn, MA 01801 USA; 4 Secretariat of the Convention On Biological Diversity, 413 Saint-Jacques Street, Suite 800, Montreal, QC H2Y 1N9 Canada; 5 United Nations Environment Programme, Pacific Office, Avele Road, Vailima, Samoa; 6 Federal Ministry of Food and Agriculture, Wilhelmstr. 54, 10117 Berlin, Germany

**Keywords:** GMO, Thesaurus, Vocabulary, Genetic elements, Genome-editing, Targeted mutagenesis

## Abstract

**Background:**

Various databases on genetically modified organisms (GMOs) exist, all with their specific focus to facilitate access to information needed for, e. g., the assistance in risk assessment, the development of detection and identification strategies or inspection and control activities. Each database has its unique approach towards the subject. Often these databases use different terminology to describe the GMOs. For adequate GMO addressing and identification and exchange of GMO-related information it is necessary to use commonly agreed upon concepts and terminology.

**Result:**

A hierarchically structured controlled vocabulary describing the genetic elements inserted into conventional GMOs, and GMOs developed by the use of gen(om)e-editing is presented: the GMO genetic element thesaurus (GMO-GET). GMO-GET can be used for GMO-related documentation, including GMO-related databases. It has initially been developed on the basis of two GMO databases, i.e. the Biosafety Clearing-House and the EUginius database.

**Conclusion:**

The use of GMO-GET will enable consistent and compatible information (harmonisation), also allowing an accurate exchange of information between the different data systems and thereby facilitating their interoperability. GMO-GET can also be used to describe genetic elements that are altered in organisms obtained through current targeted genome-editing techniques.

## Background

Since the initial advent of conventional genetically modified organisms (GMOs) [1] or—as defined by the Cartagena Protocol on Biosafety—of living modified organisms (LMOs) and products derived from GMOs on the global market, GMOs have been considered as a distinct group of new plant, animal or microbial genotypes. Consequently, GMOs and all the specific aspects related to GMOs have led to a new vocabulary describing the introduced DNA sequences, the mechanisms to achieve the gene transfer and the resulting organism harbouring the newly introduced genetic constructs.

New genome editing techniques may result in relevant alterations in an organism´s genome but may also lead to minor modifications that are indistinguishable from untargeted or spontaneous mutations in the organism’s genome. According to the European Union´s (EU) legislation, such gen(om)e-edited organisms are covered by the EU GMO legislation [2, 3]. They are considered “GE-GMO” in this paper.

**Fig. 1:**
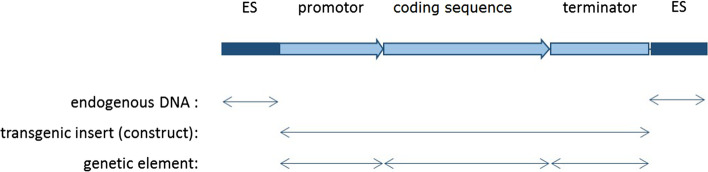
Simplified depiction of a transgenic insert in a genetically modified organism. By means of genetic engineering recombinant DNA is inserted into a genome. The recombinant DNA consists of different genetic elements that build a construct. Expression of the construct leads to manifestation of a phenotypic trait in the organism. The transgenic insert is embedded in the endogenous DNA of the organism. ES = endogenous sequence, modified from Prins et al. [24]

On the molecular level, a conventional GMO is determined by the genetic elements inserted into the endogenous DNA of the recipient organism. In case of GE-GMOs, an identifiable genetic element has been altered. The term genetic element describes a string of nucleotides (continuous DNA sequence) with a distinct function originating from one or multiple donor organism(s) or of synthetic origin, i.e. a molecular unit. Genetic elements are combined in a construct that comprises one or more coding DNA sequences (translated into proteins) or silencing elements together with appropriate regulatory DNA elements (promoters, terminators, etc.) acting during transcription or translation on DNA or RNA level to ensure correct expression of the integrated construct (Fig. 1).

Procedures for the enforcement of GMO legislation need a clear overview of the characteristics of, on the one hand, authorised GMOs that have been assessed for their safety for human and animal consumption and the environment, and of unauthorised GMOs, on the other hand, that have not (yet) been assessed by regulators of the respective countries. For an effective overview of the known GMOs, authorised and unauthorised, and also for inspection services and enforcement laboratories, it is important to have user-friendly databases providing easy access to all required information on each known GMO, being conventional or GE-GMO.

Various GMO databases have been compiled in the recent years and decades, all with their specific focus to facilitate access to information needed for, e. g., the assistance in risk assessment, the development of detection and identification strategies or inspection and control activities. A list of GMO-related websites is given in the EUginius database (https://euginius.eu/euginius/pages/intrestingLinks.jsf). Each database has its unique approach towards the subject. While each database contains unique information, there is a considerable overlap of information among them as exemplarily shown for some selected databases in Table 1. To complicate matters, these databases use different vocabularies to describe GMOs. This may lead to confusing situations, if users interpret the data differently than originally intended by the owner of the database. Another consequence is that these different vocabularies hamper an easy exchange of data between these databases. In practice, for adequate GMO identification it is essential to exchange GMO-related information and to do this effectively, it is necessary to use agreed concepts and terminology.

**Table 1:** GMO databases

Organisation	Database	Characteristics
Biosafety Clearing House (BCH)	http://bch.cbd.int/database/lmo-registry/ [25]	OECD unique identification, event, organism (food, feed), gene, trait, decision, risk assessment, country approval (world-wide)
EUginius GMO database	http://www.euginius.eu [9]	OECD unique identification, event, organism (food, feed), gene, trait, decision, risk assessment, methods, amplicon sequences, event sequences, country approval (EU, in the near future world-wide), analysis tool
Food and Agriculture Organisation (FAO)	http://www.fao.org/food/food-safety-quality/gm-foods-platform [26]	OECD unique identifier, commodity (foods), trait, country approval (world-wide); restricted to approved GM food
GM crops database (GenBit)	https://www.genbitgroup.com/en/gmo/gmodatabase/ [27]	Gene, events, trait, crop, country approval (RUS, EU)
GMO Detection Method Database (GMDD) of Shanghai University	http://gmdd.sjtu.edu.cn/ [28]	Methods, gene, events, trait, sequences, country approval (world-wide)
International Service for the Acquisition of Agri-biotech Applications (ISAAA)	http://www.isaaa.org/gmapprovaldatabase/ [29]	OECD unique identifier, event, gene, trait, crop, developer, country approval (world wide)
Joint Research Center of the European Commission (JRC) GMO Methods database	http://gmo-crl.jrc.ec.europa.eu/gmomethods [7]	Methods, gene, events (*link to BCH*), amplicon sequences, country approval (EU)
Organisation for Economic Co-operation and Development (OECD) BioTrack Product Database	http://www2.oecd.org/biotech [30]	OECD unique identifier, organism, trait, gene, first country approval (world-wide), only products approved for food, feed or environment

GMOs that are in the process of market approval and commercialisation in any member country of the Organisation for Economic Co-operation and Development (OECD), are specifically assigned with a unique identifier (UID) for unambiguous designation (not including the more recent GE-GMOs) [4, 5]. However, such a controlled vocabulary based on international consensus does not exist for the genetic elements introduced into GMOs.

**Fig. 2:**
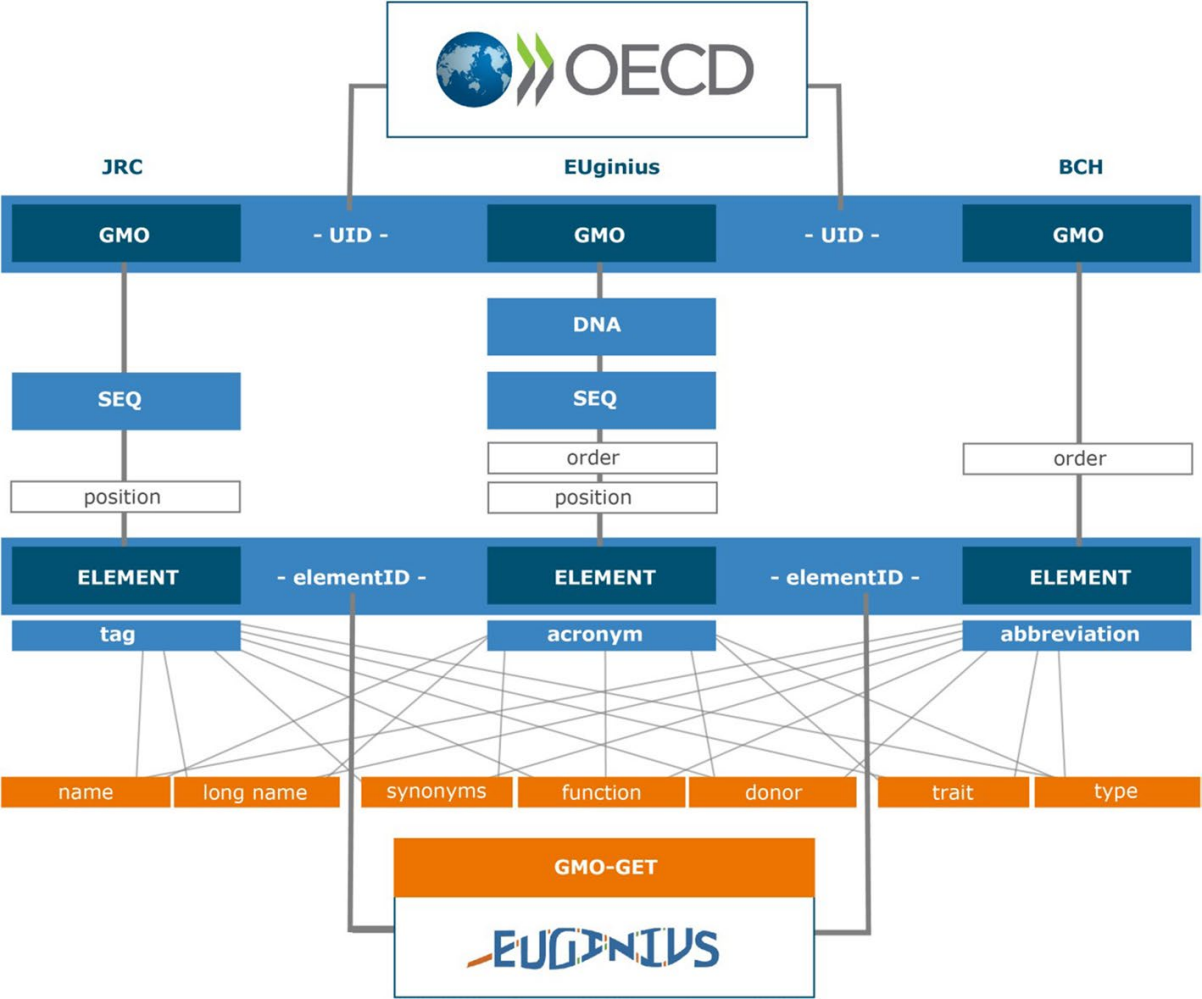
Interoperability of GMO-related databases using GMO-GET. The EUginius GMO database, BCHs’ LMO & Element Registry and JRC’s Central Core DNA Sequence Information System (CCSIS) are able to refer to each other’s data via OECD’s Unique GMO Identifier (UID) and/or via EUginius’ GMO Genetic Element Thesaurus Identifier (GMO-GET elementID). Thus, all three database systems can make use of a harmonized knowledge base identifying explicitly GMOs and their specific components described in terms of their (long) name, synonyms, function, donor, trait and type

To this end, this paper presents a hierarchically structured controlled vocabulary describing the genetic elements introduced or altered in conventional GMOs or GE-GMOs. We propose the term GMO genetic element thesaurus (GMO-GET). GMO-GET can apply to any GMO-related documentation, including any GMO-related database. It has initially been developed on the basis of three GMO databases, i.e. the database of the Biosafety Clearing-House (BCH; www.bch.cbd.int) [5], the European Database of Reference Methods for GMO Analysis (gmo-crl.jrc.ec.europa.eu/gmomethods) [6, 7] and the EUginius GMO database (www.euginius.eu) [8]. The latter is a joint development by the Dutch WFSR (Wageningen Food Safety Research, formerly RIKILT Wageningen University & Research) and the German BVL (Federal Office of Consumer Protection and Food Safety). Both the BCH and the EUginius database already apply the here proposed vocabulary. The use of GMO-GET will enable a consistent and compatible formal molecular characterisation to support an accurate exchange of information between the different data systems and thereby facilitating their interoperability (Fig. 2).

## Construction and content

Many bio-ontologies are stored at http://obo.sourceforge.net and are accepted by the scientific community as authoritative. All bio-ontologies assign an identifier (ID) for each term and these allow the archiving, storing and accessing of data in databases. Ontology IDs provide a means of exchanging data with unambiguous, shared meaning between databases, an ability known as 'semantic interoperability'.

Several bio-ontologies relate to plant genetic elements such as Gene Ontology [9], the Plant Ontology [10, 11] the Ontology of Genes and Genomes (OGG) [12], Sequence Ontology [13], and the Synthetic Biology Open Language (SBOL) [14]. However, since none of these existing ontologies are suitable for the purpose of describing GMO-related genetic elements we decided to establish a new vocabulary following a hierarchical structure, i.e. a thesaurus. The basic strategy of using an exchangeable format has been adopted from Gene Ontology, Plant Ontology and others. The software OBO-Edit is used to build and structure GMO-GET (http://oboedit.org/) [15, 16]. GMO-GET is publicly available via EUginius (www.euginius.eu/euginius/pages/gmo_genetic_elements.jsf) [17].

**Fig. 3:**
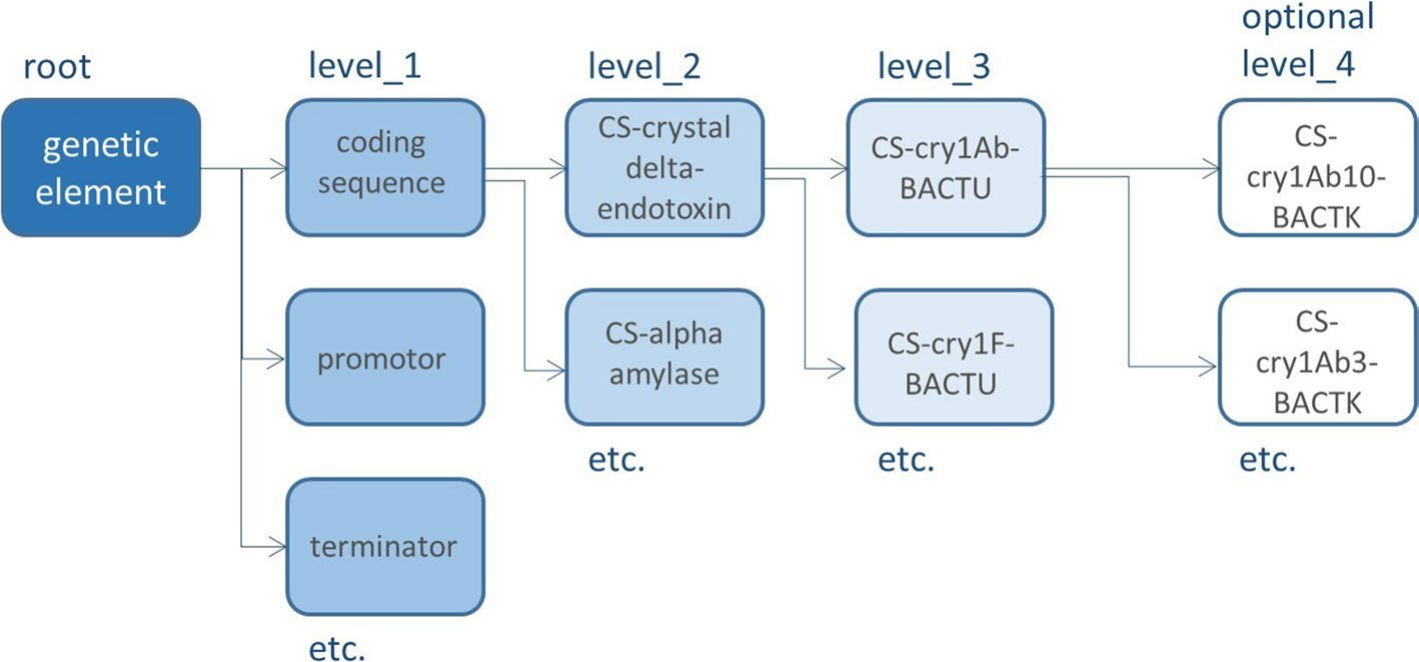
Exemplary presentation of GMO-GET. The present thesaurus is used to describe genetic elements of transgenic inserts that are found in genetically modified organisms (GMOs). The thesaurus has four levels (level_1, level_2, level_3 and level_4) that are interlinked via is_a relations, whereby one parent term can have several children terms. By means of this mono-hierarchal structure, genetic elements are classified. Level_1 describes the type of genetic element, level_2 groups genetic elements depending on their functionality, level_3 describes specific genetic elements, level_4 gives variants of a specific genetic element if there are several DNA sequences known to one element

Ontologies provide a means of formalizing knowledge in complex hierarchies that are composed of terms and rules [18, 19]. The ontology starts with a ‘root’ term, which can be connected to ‘child’ terms via defined relations. Those terms can be ‘parent’ to other terms. For example, the child-term can be related to the parent-term via an is_a relation (apple is_a fruit), or via a part_of relation (apple_peel is part of an apple). GMO-GET only uses the is_a relation (e. g. P-Cauliflower mosaic virus is_a promoter). The result is a hierarchical simple tree structure where functionally or phylogenetically related genetic elements are grouped together in branches. A set of descriptors is assigned to each element, including one preferred term precisely characterised by one definition, several synonyms (i.e. non-preferred terms), exactly one relation to a broader term and one or more relations to narrower terms as recommended in the international standard for thesauri and interoperability (ISO 25964) [20]. In addition to these ISO-descriptors, each term has its own set of properties such as an ID, a comment for scientific references and, if applicable, trait (see Table 2 for more details and Fig. 3 and Table 3 for examples). The genetic elements can, therefore, be defined in detail by means of the thesaurus, not only in terms of their individual entity but also in terms of their relation to each other. The entire tree can be seen on the page ‘List with GMOs and genetic elements’ of the EUginius website (www.euginius.eu/euginius/pages/gmo_genetic_elements.jsf) [17].

**Table 2:** Attributes describing a GMO element from the EUginius GMO genetic element thesaurus

Fieldname	Description	Example
id	Unique thesaurus ID	e: 0000415
Name	Naming convention described in text	CS-bar-STRHY
Relation	Description of type of relation and ID and name of the parent term	is_a: e: 0000188! CS-phosphinothricin N-acetyltransferase
Definition	Definition of the element, including references to the source of the information	Phosphinothricin acetyltransferase gene derived from the common soil bacterium *Streptomyces hygroscopicus* a.k.a. *bar* gene shares 85 per cent homology at the amino acid level with the pat gene. Thompson et al. (1987) EMBO J. 6:2519–2523
Comment	The function of this element, including references to the source of the information	Acetylates the primary amino group of L-phosphinothricin (L-PPT; a.k.a. glufosinate) rendering it inactive. Wehrmann et al. (1996) Nat Biotechnol. 14:1274–1278; ENV/JM/MONO(99) 13:1–26
Synonyms	Non-standard names	Bialaphos resistance, PAT
Trait	Which phenotypic trait does it influence, consists of link to a trait thesaurus (the EUginius trait thesaurus)	xref: Trait:t\:0000006 Herbicide tolerance > Glufosinate tolerance
Other_ids	Relevant IDs from BCH, JRC or other databases	xref: BCH:14972 xref: Donor: *Streptomyces hygroscopicus*

**Table 3:** Examples of GMO element thesaurus terms

Prefix	Level 2 (long name)	Level 3 (name)	Level 4	Synonyms	Donor species	Trait	Definition	Function	BCH ID
CS	CS-5-enolpyruvylshikimate-3-phosphate synthase	CS-CP4epsps-RHIRD	–	aroA	*Agrobacterium tumefaciens ssp. CP4*	t: 0000030 (Glyphosate tolerance)	5-Enolpyruvylshikimate-3-phosphate synthase gene from *Agrobacterium* sp. strain CP4. Steinrücken et al. (1980) Biochem Biophys Res Com. 94:1207–1212	Is similar and functionally identical to endogenous plant EPSPS enzymes but has a much-reduced affinity for glyphosate relative to endogenous plant EPSPS. Padgette et al. (1996) J Nutr 126:728–740	14979
CS	CS-crystal delta-endotoxin	CS-cry1Ab_vip3H-SYNTH		HJC-1	Synthetic	t: 0000035 (Lepidoptera resistance)	Chimeric gene encoding for the fused protein of Cry1Ab/Vip3H	Confers resistance to lepidopteran such as the Asiatic rice borer *Chilo suppressalis* and the stem borer *Sesamia inferens*	
CS	CS-crystal delta-endotoxin	CS-cry1Ab-BACTU	CS-cry1Ab10-BACTK	cry1Ab	*Bacillus thuringiensis*	t: 0000035 (Lepidoptera resistance)	Plant optimized gene encoding for the nature identical (full length) *cry1Ab* gene of *B. thuringiensis* ssp. Kurstaki HD-1 strain; Fischhoff D et al. (1987) Bio/Technology, 5, 807–813 (Accession no. A29125)	The trypsin resistant core of the encoded protein is insecticidal to lepidopteran larvae. It acts by selectively binding to specific sites localized on the lining of the midgut of susceptible insect species. Confers resistance against certain lepidopteran insect pests, including the European Corn Borer (ECB) (*Ostrinia nubilalis*) and pink borers (*Sesamia* spp.)	14985
CS	CS-crystal delta-endotoxin	CS-cry1Ac-BACTU		cry1Ac	*Bacillus thuringiensis ssp. Kurstaki*	t: 0000035 (Lepidoptera resistance)		The trypsin resistant core of the encoded protein is insecticidal to lepidopteran larvae. It acts by selectively binding to specific sites localized on the lining of the midgut of susceptible insect species	14986
CS	CS-phosphinothricin N-acetyltransferase	CS-bar-STRHY		Bialaphos resistance, PAT	*Streptomyces hygroscopicus*	t: 0000029 (Glufosinate tolerance)	Phosphinothricin acetyltransferase gene derived from the common soil bacterium *Streptomyces hygroscopicus* a.k.a. *bar* gene; shares 85 per cent homology at the amino acid level with the pat gene. Thompson et al. (1987) EMBO J. 6:2519–2523	Acetylates the primary amino group of L-phosphinothricin (L-PPT; a.k.a. glufosinate) rendering it inactive. Wehrmann et al. (1996) Nat Biotechnol. 14:1274–1278; ENV/JM/MONO(99) 13:1–26	14972
CS	CS-phosphinothricin N-acetyltransferase	CS-pat-STRVR		Bialaphos resistance	*Streptomyces viridochromogenes*	t: 0000029 (Glufosinate tolerance)	Phosphinothricin acetyltransferase gene derived from *Streptomyces viridochromogenes* a.k.a. *pat* gene; shares 85% homology at the amino acid level with the *bar* gene. Wohlleben et al. (1988) Gene 70:25–37	Acetylates the primary amino group of L-phosphinothricin (L-PPT; a.k.a. glufosinate) rendering it inactive. Wehrmann et al. (1996) Nat Biotechnol.14:1274–1278; ENV/JM/MONO(99) 13:1–26	15002
CS	CS- Polyphenol oxidase	CS-ppo5_genome_edited-SOLTU			*Solanum tuberosum*	t: 0000055 (Reduced black spot bruising)	Polyphenol oxidase 5 gene. Thygesen et al. (1995) Plant Physiol. 109:525–531	Active in tubers, major cause of enzymatic browning. Loss-of-function mutations in the ppo5 gene result in reduced browning of potato tubers (reduced 'black spot')	
CS	CS- Polyphenol oxidase	CS-ppo_genome_edited-AGABB			*Agaricus bisporus*	t: 0000029 (Glufosinate tolerance)	Polyphenol oxidase gene. Wu et al. (2010) Biotechnol Letters 32(10):1439–1447	Major cause of enzymatic browning. Loss-of-function mutations in the *ppo* gene leads to reduced enzymatic browning	
P	P-34S FMV P-Figwort mosaic virus	P-34S FMV		P-35SFMV, P-CMoVb	*Figwort mosaic virus*		34S promoter derived from Figwort mosaic virus (FMV). Shepard et al. (1987) Phytopathology 77:1668–1673; Richins et al. (1987); Nucl Acids Res. 15:8451–8466; Gowda et al. (1989) J Cell Biochem. 13D (supplement):301	Promoter (directs transcription)	101507
P	P-35S CaMVP-Cauliflower mosaic virus	P-35S-CaMV		P-35S, P-CaMV 35S	*Cauliflower mosaic virus*		The 35S promoter was isolated from the Cauliflower mosaic virus (CaMV). The element covers the full-length promoter as well as optimized variants of the promoter. Odell JT et al. (1985) Nature 313, 810–812	The 35S promoter is a very strong constitutive promoter, resulting in high levels of gene expression in dicot plants. However, it is less effective in monocots, especially in cereals	100287
P	P-nopaline synthase	P-nos-RHIRD		pNOS	*Agrobacterium tumefaciens*		Promoter region of the nopaline synthase gene from *Agrobacterium tumefaciens* T-DNA. Bevan et al. (1983) Nucl Acids Res. 11:369–385; Fraley et al. (1983) Proc Nat L Acad Sci. 80:4803–4807	Promoter (directs transcription)	100270
P	P-ubiquitin	P-ubi1-MAIZE		P-ZmUbi1, P-ubiZM1	*Zea mays*		Promoter region of the polyubiquitin gene from *Zea mays*. Christensen et al. (1992) Plant Mol Biol. 18: 675–689	Promoter (directs transcription)	100362
T	T-35S CaMV T-Cauliflower mosaic virus	T-35S-CaMV		3′ 35S, 35S TERM	*Cauliflower mosaic virus*		3′ Transcriptional termination element (3′ UTR) of the 35S gene from Cauliflower mosaic virus. Gardner et al. (1981) Nucleic Acids Res. 9:2871–2888	Terminator (indicates the end of transcription; directs polyadenylation)	100290
T	T-nopaline synthase	T-nos-RHIRD		3′ nos, NOST	*Agrobacterium tumefaciens*		3′ Transcriptional termination element (3′ UTR) of the nopaline synthase gene from *Agrobacterium tumefaciens* T-DNA. Bevan et al. (1983) Nucleic Acids Res. 11: 369–385; Fraley et al. (1983) Proc Nat L Acad Sci. 80:4803–4807	Terminator (indicates the end of transcription; directs polyadenylation)	100269
T	T-RuBisCO small subunit	T-rbcS_E9-PEA		T-RuBisCO SSU, T-SSU, T-rbcS-E9	*Pisum sativum*		3′ Transcriptional termination element (3′ UTR) of the rinulose-1,5-bisphosphate carboxylase, small subunit (*rbcS E9*) gene. Coruzzi et al. (1984) EMBO J. 3:1671–1679	Terminator (indicates the end of transcription; directs polyadenylation)	101877

### GMO-GET is structured in five hierarchical levels

Level_0 is the root representing the general idea of a genetic element. Attached to the root are the level_1 terms. Level_1 describes the general functional types of genetic elements: coding sequence, enhancer, gene silencing elements, genomic sequence, intron, leader, promoter, other regulatory elements, terminator, transit peptide, unknown origin, vector fragment. The lower level (level_2) is composed of an abbreviation of the element type plus a generally comprehensive long name referring to the detailed biological function, e.g. CS-nopaline synthase. Below level_2, genetic elements of common/homologous origin (e.g. *cry* delta endotoxin genes) and/or with comparable/analogous features (e.g. variants of the *epsps* gene) are grouped. Those level_3 terms serve as a label for the actual genetic elements and are described with an abbreviation following specific syntax rules. Therefore, each genetic element listed in GMO-GET has an unambiguous designation (e.g. P-nos-RHIRD).

Where information is available a fourth level, level_4, can be added to collect variants of a genetic element with, e.g., minor sequence differences resulting from cloning strategies, spontaneous or induced mutagenesis. In allopolyploid GE-GMOs it could define variants of differently modified homoeologs. Such records in level_4 can be used to assign methods that target specific variants of a genetic element and, vice versa, exclude genetic elements that do not contain the target sequence of a method.

The general syntax for a genetic element in level_3 of GMO-GET is XX-YYYY-ZZZZZ, with a prefix (XX), a name part (YYYY) and a donor part (ZZZZZ). For gene silencing elements and elements modified using genome editing techniques, the name part also includes information about the particularity of the elements as a suffix after YYYY. For example, YYYY_genome_edited indicates an element modified by the use of genome-editing techniques, YYYY_siRNAs indicates a sense orientation of elements leading to gene silencing through siRNA, YYYY_siRNAas an antisense orientation and YYYY_siRNAu the undefined orientation of this element.

The prefix (XX, one or two characters) indicates the element type, i.e. whether the genetic element is a coding sequence (CS-), an intron (I-), a promotor (P-) or something else. All currently used prefixes are listed in Table 4.

**Table 4:** Prefixes for GMO elements

Prefix	Element type	Prefix	Element type
CS	Coding sequence	P	Promoter
E	Enhancer	R	Regulatory element other than P, T, I, L or TP
I	Intron	T	Terminator
L	Leader	TP	Transit peptide
O	Other sequence	V	Vector fragment

The middle part of the syntax (YYYY, without fixed length) accounts for an abbreviation of the element. The abbreviation should reflect the most common abbreviation of the element. If an abbreviation does not exist or a common abbreviation is difficult to pinpoint, an easy-to-understand abbreviation of the element name should be used upon agreement by the constructors of the thesaurus. The abbreviation should be the same for all types of an element (i.e. promoter, terminator, etc.). Some rules were established to design the middle part of the syntax:

1. The abbreviation of the element name is written in lowercase letters unless the commonly used abbreviation uses uppercase letters. If the common abbreviation includes a species abbreviation, this should be deleted from the name since this will be indicated in the suffix of the element name (e. g. CS-AtAHAS will translate into CS-ahas-ARATH, P-ZmUbi1 will translate into P-ubi1-MAIZE); an exception from this rule is the naming of the *Agrobacterium tumefaciens* (update scientific name *Rhizobium radiobacter*) strain CP4. As there is no specific abbreviation for this strain “CP4” will be part of -YYYY-, e.g. CS-CP4epsps-RHIRD.
2. Special characters are avoided as much as possible and are exchanged by an underline “_”. E.g. “.”, “/” and “´” are interpreted as special characters.
3. Greek symbols are written out instead of using the one letter code (e.g. CS-beta-gal, instead of β).
4. The 3′ UTR and 5′ UTR (untranslated region) should be removed from element names since this seems obvious from the fact that the element is a terminator (T-) or promoter (P-).

Finally, for conventional GMOs, the syntax includes an organism code as suffix or donor part (ZZZZZ, usually four or five characters), which denominates the donor species or source of the genetic element by giving an abbreviation for the species in capital letters; hybrid elements have the donor SYNTH. The organism code follows the recommendations suggested by UniProt (http://www.uniprot.org/docs/speclist) [21] except for viruses. Abbreviations to reflect species of viruses are adopted from Plant Viruses Online—Index to Virus Acronyms (http://bio-mirror.im.ac.cn/mirrors/pvo/vide/acrindex.htm) or, if not found there, from *the Ninth Report of the International Committee on Taxonomy of Viruses (2012)* [22].

## Utility and discussion

Genetic elements are used to identify a conventional GMO, i.e. by describing the genetic elements that are present, either as part of the insert or as endogenous (flanking of species-specific) sequence, a conventional GMO can be identified unambiguously. Therefore, GMO-GET enables the precise description of a conventional GMO by its genetic elements and the thesaurus makes it possible to integrate data from databases which use these terms even if from different hierarchical levels. For example, the BCH annotates GMOs in its databases with genetic elements that are defined with two terms, name and abbreviation, but without hierarchy. EUginius adopted those terms and incorporated them into GMO-GET: The BCH name became level_2 and the BCH abbreviation is level_3. EUginius uses level_3 terms of GMO-GET to describe GMOs and uses the hierarchical structure of GMO-GET to allow identification of related genetic elements and subsequently corresponding GMOs and/or methods. When the hierarchy of the thesaurus is used, a query with a level_2 term will find the same GMOs (if present) in other databases using GMO-GET, because the query will also find GMOs annotated with children (level_3 and level_4) of this particular level_2 term.

The EUginius database uses GMO-GET in its approach to provide major and relevant information on GMOs. This database is based on four interconnected modules. (1) The GMO module lists existing conventional GMOs and genome edited organisms and enables sorting and filtering by specific criteria like trait, company or genetic elements. It also provides detailed information on the molecular characterisation including annotated sequences. (2) The detection module contains information on detection methods including reference materials, tools supporting the development of screening strategies and relevant literature. (3) The analysis module provides a tool for the interpretation of screening test results. (4) The authorisation module offers detailed authorisation status and EU application details on food and feed.

GMO-GET is used (1) in the GMO module to describe the inserts in GMOs, and (2) especially for identifying non authorized GMOs: It is the structural basis for the GMO method matrix of the detection module and the analysis module.

By using GMO-GET, EUginius offers a way to include all information available on GE-GMOs—including trait, genetic element affected, developer, etc.—in a structured and standardized way. The standardisation makes the information on GE-GMOs available to systems that support inspection and control activities.

Based on the dual entity relation of many genetic elements it is also possible to use a computer to predict which GMO can be detected with a particular element-specific method. No manual intervention is required. EUginius uses this information, for example, for its Detection module and Analysis module, which facilitate the selection of appropriate detection methods and the interpretation of corresponding results of GMO analysis experiments.

## Conclusions

The genetic element thesaurus GMO-GET presented here is the first ontology that provides a controlled vocabulary for GMO-derived genetic elements using an unambiguous and harmonised vocabulary with a predictable but flexible set of syntax rules. The thesaurus can easily be expanded, if needed. GMO-GET thereby allows exchange of information between databases with overlapping information by increasing the possibilities for automatic data exchange between databases. Only the element ID (or element name) needs to be transferred as the properties of the element are already defined in GMO-GET for the linked databases. Thus, linkage of databases using GMO-GET will enable supplementing the information from one database with additional information from the other database(s). Thereby the user of the databases does not have to perform a new, modified, query for each database, but can rely on asking for information on the same genetic element in all linked databases. The GMO-GET thesaurus with syntax rules and clear hierarchy enables the extrapolation of new terms from different parties without lengthy discussions on individual terms.

GMO-GET is the only known system that addresses organisms derived through gen(om)e-editing techniques, which are considered GMO in the EU but not necessarily everywhere else in the world.

Furthermore, GMO-GET allows explicit assignment of PCR methods for detection of genetic elements and of corresponding GMOs that contain the DNA sequence of the construct used for transformation. Since GMO-GET also offers information on the relationship of the genetic elements, using the hierarchical properties of the thesaurus, this also enables linking properties of one genetic element to another, related, genetic element. This increases the investigative potential of existing PCR detection methods by linking them not only to GMOs containing the confirmed target elements but also to GMOs containing ‘children’ of those elements. It names the modified gene of GE-GMO and supports the development of specific detection methods.

GMO-GET will thus facilitate harmonised enforcement strategies based on a clear overview of the characteristics of known authorised as well as unauthorised GMOs.

## Data Availability

The GMO-GET is available via the website www.euginius.eu. The thesaurus is also available in OBO-format and can be requested from the corresponding authors on reasonable request.

## Abbreviations

BCH: Biosafety Clearing-House
EU: European Union
GMO: Genetically modified organism
GE-GMO: Gen(om)e-edited genetically modified organism
GMO-GET: Genetically modified organism genetic element thesaurus
ID: Identifier
LMO: Living modified organism
PCR: Polymerase chain reaction
UID: Unique IDentifier
